# Co-expression of MDM2 and CDK4 in transformed human mesenchymal stem cells causes high-grade sarcoma with a dedifferentiated liposarcoma-like morphology

**DOI:** 10.1038/s41374-019-0263-4

**Published:** 2019-06-03

**Authors:** Yu Jin Kim, Mingi Kim, Hyung Kyu Park, Dan Bi Yu, Kyungsoo Jung, Kyoung Song, Yoon-La Choi

**Affiliations:** 1 Laboratory of Cancer Genomics and Molecular Pathology, Samsung Medical Center, Sungkyunkwan University School of Medicine, Seoul, Korea; 2 Department of Health Sciences and Technology, SAIHST, Sungkyunkwan University, Seoul, Korea; 3 Department of Pathology, Konkuk University Medical Center, Konkuk University School of Medicine, Seoul, Korea; 4 The Center for Companion Diagnostics, LOGONE Bio Convergence Research Foundation, Seoul, Korea; 5 Department of Pathology and Translational Genomics, Samsung Medical Center, Sungkyunkwan University School of Medicine, Seoul, Korea

**Keywords:** Sarcoma, Tumour biomarkers

## Abstract

Amplification and overexpression of MDM2 and CDK4 are well-known diagnostic criteria for well-differentiated liposarcoma (WDLPS)/dedifferentiated liposarcoma (DDLPS). Although it was reported that the depletion of *MDM2* or *CDK4* decreased proliferation in DDLPS cell lines, whether MDM2 and CDK4 induce WDLPS/DDLPS tumorigenesis remains unclear. We examined whether MDM2 and/or CDK4 cause WDLPS/DDLPS, using two types of transformed human bone marrow stem cells (BMSCs), 2H and 5H, with five oncogenic hits (overexpression of hTERT, TP53 degradation, RB inactivation, c-MYC stabilization, and overexpression of HRAS^v12^). In vitro functional experiments revealed that the co-overexpression of MDM2 and CDK4 plays a key role in tumorigenesis by increasing cell growth and migration and inhibiting adipogenic differentiation potency when compared with the sole expression of MDM2 or CDK4. Using mouse xenograft models, we found that the co-overexpression of MDM2 and CDK4 in 5H cells with five additional oncogenic mutations can cause proliferative sarcoma with a DDLPS-like morphology in vivo. Our results suggest that the co-overexpression of MDM2 and CDK4, along with multiple genetic factors, increases the tendency for high-grade sarcoma with a DDLPS-like morphology in transformed human BMSCs by accelerating their growth and migration and blocking their adipogenic potential.

## Abstract (web summary)

This paper examines whether the nuclear E3 ubiquitin ligase MDM2 and/or the cyclin-dependent kinase CDK4 produce well-differentiated liposarcoma (WDLPS)/dedifferentiated liposarcoma (DDLPS) using transformed human bone marrow stem cells through in vitro and in vivo functional experiments. The authors found that co-overexpression of MDM2 and CDK4 causes high-grade sarcoma with a dedifferentiated liposarcoma-like morphology.

## Introduction

Liposarcoma (LPS) is one of the most frequently occurring types of soft tissue sarcoma in adults [1]. According to its histological characteristics, LPS consists of three categories: well-differentiated or dedifferentiated, myxoid/round cell, and pleomorphic LPSs [1]. Well-differentiated (WD) or dedifferentiated (DD) LPS is the most common subtype and is associated with supernumerary ring and/or giant rod chromosomes formed by the amplification of chromosome 12q13-15, which contains several hundred genes, including *MDM2* and *CDK4* [2]. Amplification and overexpression of MDM2 and CDK4 are generally accepted as the current diagnostic criteria for WDLPS/DDLPS [3–5].

MDM2 inhibits tumor suppressor p53 and is overexpressed in numerous cancers [6]. MDM2 functions as a ubiquitin ligase that targets p53 through the proteasomal degradation pathway; it also participates in its own autodegradation to prohibit MDM2 activity, inhibiting p53 during periods of cellular stress [7, 8]. CDK4 forms a complex with cyclin D, which then phosphorylates pRB. This prevents E2F from interacting with phosphorylated pRB, which causes the cell cycle to progress into the G1–S transition and increases cell proliferation [9–11]. Knockdown of *MDM2* or *CDK4* decreased cell proliferation in DDLPS cells [4]. Despite their potency as driving factors, whether MDM2 and CDK4 induce WDLPS/DDLPS tumorigenesis remains unclear.

It has been well-established that the genetic manipulation of important tumor suppressor genes and oncogenes induces the transformation of human BMSCs to various sarcomas in vitro or in vivo [12–16]. However, studies have failed to model sarcomagenesis through the expression of fusion oncogenes in human mesenchymal stem cells (MSCs) [17, 18]. Recently, robust evidence has shown that the expression of the FUS-CHOP fusion protein may initiate myxoid liposarcoma in transformed human BMSCs containing five different oncogenic mutations [19]. After conducting a systemic literature review, we found that these genetic events were not directly relevant to WDLPS/DDLPS. Therefore, we chose to use the transformed human BMSCs to examine whether MDM2 and/or CDK4 uniquely cause WDLPS/DDLPS.

Binh et al. found that the immunoexpression of MDM2 or CDK4, MDM2, and CDK4 was 100% (44/44), 90.9% (40/44), and 90.9% (40/44) in WDLPS and 95.1% (58/61), 91.8% (56/61), and 90.2% (55/61) in DDLPS, respectively [20]. Sirvent et al. reported that the immunopositivities of MDM2 and CDK4 were 76.5% (26/34) and 82.4% (28/34) in WDLPS, and 100% (8/8) and 100% (8/8) in DDLPS, respectively [21]. In our previous study, the amplifications of *MDM2* and *CDK4* were 100% (30/30) and 90% (27/30) in WDLPS, and 100% (26/26) and 92.3% (24/26) in DDLPS, respectively [22]. Based on the overexpression of both MDM2 and CDK4 in WDLPS/DDLPS, using transformed human BMSCs, we examined whether the co-overexpression of MDM2 and CDK4 drives WDLPS/DDLPS tumorigenesis.

## Materials and methods

### Cell lines and reagents

Transformed 2H and 5H human bone marrow stem cells (BMSCs) and the LIPO-863B and LP6 cell lines were kindly provided by Dr. Pablo Menendez, Dina Lev, and Jonathan A Fletcher, respectively [17, 23, 24]. The cells were cultured in the Dulbecco’s Modified Eagle Medium (DMEM) (Thermo Fisher Scientific, Waltham, MA, USA) containing 10% fetal bovine serum (FBS) (Gibco, Grand Island, NY, USA) and 1% antibiotic–antimycotic solution (Gibco) at 37 °C and in 5% CO_2_ conditions. Mycoplasma contamination was not detected in any of the cell cultures.

### qRT-PCR

cDNA was generated from the total RNA using SuperScript III Transcriptase, according to the manufacturer’s instructions (Invitrogen, Carlsbad, CA, USA). Quantitative reverse transcription (qRT)-polymerase chain reaction (PCR) amplification of stemness- or adipogenesis-related genes was performed using probes and primers with the Universal Probe Library System (Roche, Basel, Switzerland). For *MDM2*, the following primer pair was used: forward, (5′-ACCTCACAGATTCCAGCTTCG-3′); reverse, (5′-TTTCATAGTATAAGTGTCTTTTT-3′). *HPRT1* was used as a reference gene, and the ratio of the expression of each gene to that of *HPRT1* was calculated for the relative quantification of the expression level of each gene. To determine the mRNA levels of *hTERT*, *E6*, E7, *small t*, *HRAS*^*v12*^, and TP53, qRT-PCR was performed using SYBR Green PCR Master Mix (Applied Biosystems) and specific primer sets (Supplementary Table 1).

### Immunoblotting, immunocytochemistry, immunohistochemistry, and fluorescence-activated cell sorting (FACS)

Equal amounts of protein were subjected to SDS-PAGE on an 8.5% gel before being transferred to a nitrocellulose membrane (Pall Corporation). The membrane was incubated with primary anti-MDM2, CDK4, and β-actin (diluted 1:1000 in 5% nonfat milk, Santa Cruz Biotechnology) and FLAG (diluted 1:1000 in 5% nonfat milk, Sigma-Aldrich) antibodies, and then washed (for 30 min) with T-BST. The membrane was then incubated with horseradish peroxidase-conjugated secondary goat anti-rabbit or anti-mouse antibodies (diluted 1:2000 in 5% nonfat milk, Abcam) for 1 h, followed by 30 min of washing with T-BST. Signals were detected using ECL solution (Thermo Fisher Scientific). Four-micrometer-thick sections from formalin-fixed paraffin-embedded cell or tissue blocks were cut with a microtome and routinely deparaffinized. The antigen retrieval procedure was performed in 0.01 M of citrate buffer (pH 6.0) at 95 °C, and counterstaining was performed using hematoxylin. The anti-MDM2 (Invitrogen, IF2, 1:200 dilution), CDK4 (Invitrogen, DCS-31, 1:50 dilution), and KU80 (Cell Signaling; C48E7, 1:200 dilution) antibodies were used for immunocytochemical or immunohistochemical staining using the automated bench-mark XT platform (Ventana Medical Systems). The cells were washed with FACS buffer (PBS, 0.5% BSA, 0.1% NaN_3_ sodium azide) and stained with anti-CD34 and CD105 (BD Biosciences) antibodies. Isotype-matched FITC/PE-conjugated controls were also included with each set. The positive cells were analyzed by BD FACS Verse flow cytometry (BD Biosciences).

### Generation of stable cell lines overexpressing MDM2 and/or CDK4

The full-length cDNAs of *MDM2* or *CDK4* were generated from a cDNA library of human BMSCs. The PCR products were cloned into the N-terminal p3XFLAG-CMV-10 vector (Sigma-Aldrich). We confirmed the full sequence of wild-type *MDM2* and *CDK4* by the Sanger sequencing method. Full-length 3XFLAG-*MDM2* or 3XFLAG-*CDK4* was cloned into the gateway entry vector pCR8/GW/Topo (Invitrogen), and then subcloned into pLenti6.3/V5-DEST (Invitrogen). Full-length sequences of 3XFLAG-*MDM2* or 3XFLAG-*CDK4* were validated by Sanger sequencing. pLenti6.3/3XFLAG-*MDM2* or 3XFLAG-*CDK4* expression vectors were transfected into 293FT cells using ViraPower Packaging Mix (Invitrogen) to produce lentiviruses expressing MDM2 or CDK4. After 48 h, lentiviral supernatants were harvested and transduced into 2H and 5H cells in the presence of 8 µg/mL of polybrene. The transduced cells were grown in DMEM complete medium for 48 h after infection, and then, the medium was replaced with medium containing blasticidin (5 µg/mL) after 24 h. The cells were then seeded into 96-well plates at a density of one cell/well in selective medium for 2 weeks. Live cell clones were checked using microscopy. These colonies were subcultured into 24- or 6-well plates. Stable expression of MDM2 or CDK4 was confirmed by qRT-PCR and immunoblotting.

### Cell proliferation and migration assays and soft agar assay

The cell proliferation assay was performed using an EZ-CYTOX kit (Daeil Lab Service), according to the manufacturer’s instructions. Cells were plated in 96-well plates (3 × 10^2^ cells/well). The 96-well plates were incubated with EZ-CYTOX reagent for 3 h at 37 °C after 1 and 2 days. Twenty-four-well transwell chambers (Corning Costar) with 8-μm polycarbonate membrane filters were used to determine the migration ability. For this assay, 5 × 10^4^ cells were seeded into the upper chamber in the DMEM without FBS. The lower chamber contained 700 μL of the DMEM containing 10% FBS. The transwell chamber was incubated at 37 °C in 5% CO_2_ conditions. After 24 h of incubation, the non-migrating cells on the upper filter surface were removed with a cotton swab and the migrated cells were stained with 0.5% crystal violet. The cells were then seeded into 24-well plates with the appropriate concentrations of agarose (0.5% for base and 0.3% for top) to form colonies in 3 weeks. The colonies were stained with crystal violet (0.5% w/v) and counted using a microscope.

### Adipogenic differentiation

BMSCs and the 2H and 5H cells were seeded onto a six-well plate in the DMEM medium; the medium was replaced with adipogenic differentiation medium (StemPro Adipogenic Differentiation Kit, Invitrogen) every 3–4 days. After 21 days, the cells were stained with an Oil Red O staining kit (Lifeline) according to the manufacturer’s instructions.

### Mouse xenograft modeling

This study was reviewed and approved by the Institutional Animal Care and Use Committee of the Samsung Biomedical Research Institute (SBRI, Seoul, Korea). SBRI is an Association for the Assessment and Accreditation of Laboratory Animal Care International accredited facility and abides by the Institute of Laboratory Animal Resources guide (No. 20160108001). Female nude mice were injected subcutaneously with 2H, 5H, LIPO-863B, or LP6 (5 × 10^6^) cells. After the indicated number of days, tumor diameter was measured using a digital caliper two or three times per week, and tumor sizes were estimated using the following formula: (3.14/6) (length × width^2^).

## Results

### Transformed human BMSCs retain their stemness characteristics

**Fig. 1:**
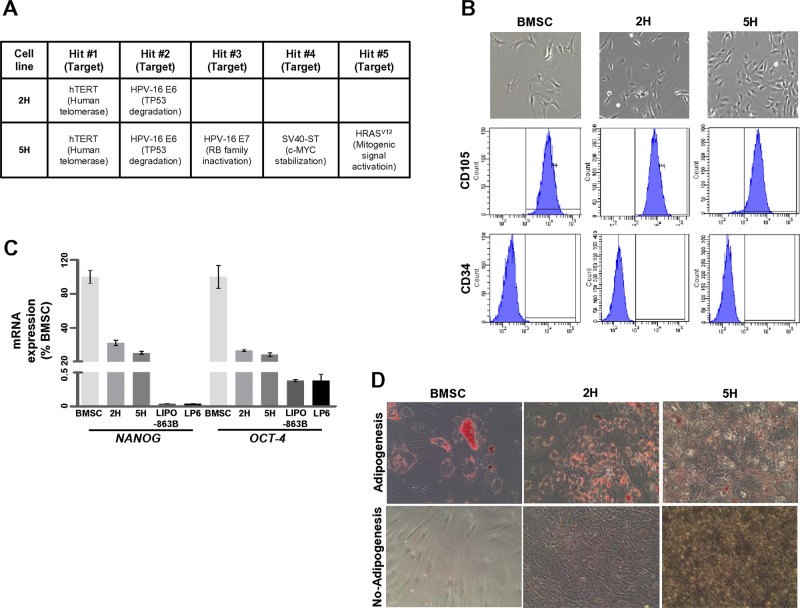
Transformed human BMSCs retain their stemness characteristics. **a** The oncogenic hits used in the 2H and 5H cells are indicated. **b** 2H and 5H cells underwent morphologic changes in vitro, adopting shorter and thicker appearances than BMSCs (upper panels). Expression of two cell surface makers (CD105 and CD34) was validated by fluorescence-activated cell sorting (FACS) analyses based on isotype-matched control antibodies (lower panels). **c** mRNA expression levels of *NANOG* and *OCT-4* were quantified using quantitative RT-PCR after the induction of adipogenesis for 21 days. The percentage values were calculated based on their levels in BMSCs. **d** The ability to differentiate into adipocytes was monitored by Oil Red O staining

To examine whether the co-overexpression of MDM2 and CDK4 drives WDLPS/DDLPS tumorigenesis, we used two types of transformed BMSCs (2H and 5H cells) containing two to five different oncogenic mutations (Fig. 1a) [19]. These oncogenic hits include the following: (i) ectopic overexpression of human telomerase reverse transcriptase (hTERT), (ii) TP53 degradation by the expression of the E6 antigen of human papillomavirus-16 (HPV-16), (iii) RB family inactivation by the expression of the E7 antigen of HPV-16, (iv) c-MYC stabilization by the expression of the small T antigen of Simian virus 40 (SV40), and (v) activation of mitogenic signal by the expression of HRAS^v12^. The E6 antigen of HPV-16 mediates TP53 degradation via the proteasomal degradation pathway, as observed in the case of MDM2. However, E6 and MDM2 are regulated through well-established distinct mechanisms [25, 26]. Therefore, none of the five oncogenic aspects were directly relevant to WDLPS/DDLPS.

We examined the characteristics of 2H and 5H cells and compared them with those of non-transformed BMSCs. The 2H and 5H cells expressed cell surface makers, such as CD105 (human mesenchymal stromal cell marker; positive) and CD34 (hematopoietic progenitor cell marker; negative), which is consistent with BMSCs (Fig. 1b). However, the morphology of 2H and 5H cells differed from that of BMSCs, which are characterized by a spindle-shaped morphology, which includes a large cell body with long and thin tails. Both 2H and 5H cells were shorter and thicker than BMSCs, while 5H cells were much shorter and more radial than 2H cells. To evaluate expression levels of stemness genes in 2H and 5H cells, we compared the expression in these cells with that in two representative liposarcoma cell lines, LIPO-863B (WDLPS) and LP6 (DDLPS). The 2H and 5H cells showed sustained expression of *NANOG* and *OCT-4* mRNA in the presence of an adipogenesis-inducing medium; however, the expression of these genes in 5H cells was lower than that in 2H cells (Fig. 1c). In addition, the 2H and 5H cells showed upregulation of *NANOG* and *OCT-4* only when cultured in the DMEM (Supplementary Fig. 1). 2H and 5H cells were maintained in a manner in which they retained their ability to differentiate into adipocytes in response to adipogenesis medium, despite the low potency rate, relative to the BMSCs (Fig. 1d). These findings suggest that the 2H and 5H cells retained the characteristics of BMSCs.

### Co-overexpression of MDM2 and CDK4 synergistically drives tumorigenic phenotypes of transformed human BMSCs

**Fig. 2:**
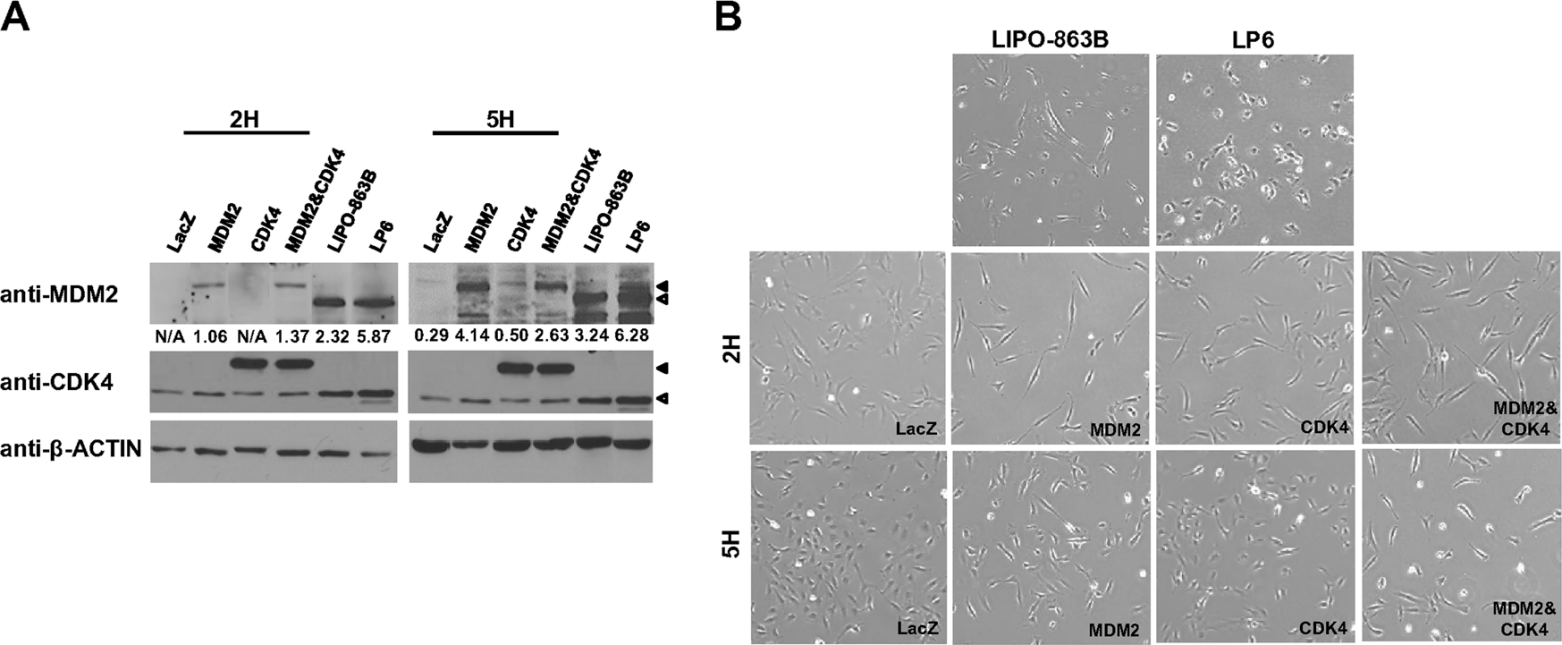
2H and 5H cells stably express MDM2 and/or CDK4 and display morphologic changes. **a** The expression of proteins of MDM2 and/or CDK4 was measured by immunoblotting. ◀, 3XFLAG-MDM2 or CDK4; ◁, MDM2 or CDK4. Fold changes were determined by comparing the protein levels to the β-actin levels using ImageJ. **b** Morphologic changes were assessed by the comparison of protein expression in different cell lines

To establish cells co-overexpressing MDM2 and CDK4, 2H and 5H cells were infected with either LacZ- (β-galactosidase, control) or MDM2- and/or CDK4-expresssing lentiviral particles. Expression of the mRNA transcript and protein of MDM2 and/or CDK4 was confirmed by immunoblotting (Fig. 2a) and qRT-PCR (Supplementary Fig. 2). To evaluate the expression levels of MDM2 and/or CDK4 in transduced 2H and 5H cells, we compared the expression in these cells with that in LIPO-863B and LP6 cell lines. The MDM2 protein expression increased by 1.06–4.14 fold in 2H and 5H cells, respectively, and the fold increase was 2.32–3.24 for LIPO-863B cells. In addition, *MDM2* mRNA expression values exhibited fold changes of 0.29 or 0.62 in 2H and 5H cells, respectively, when compared with the expression in LIPO-863B cells (Supplementary Fig. 2). Therefore, both MDM2 protein and mRNA expression values in 2H and 5H cells were observed at biological levels. CDK4 expression in the transduced 2H and 5H cells was more than twofold higher than that in both LP6 and LIPO-863B cells (Fig. 2a). Morphologically, both 2H and 5H cells co-overexpressing MDM2 and CKD4 were much longer and thinner than those solely expressing MDM2, CDK4, or LacZ (Fig. 2b; Supplementary Fig. 3). Because all these cell lines were generated from single-cell clones, we confirmed the expression of the five oncogenic hits using qRT-PCR (Supplementary Fig. 3). The *hTERT* and *E6* expression levels were notably high in both the 2H and 5H cells; the expression of E7 and *small t* mRNA was notably high in the 5H cells (Supplementary Fig. 3). In addition, the expression of *HRAS*^*v12*^ in the 5H and 2H cells showed no significant difference (Supplementary Fig. 4).

HPV-16 E6 promotes TP53 ubiquitination and degradation [27, 28]. To evaluate the TP53 expression levels in the established cells expressing MDM2 and/or CDK4 that had been transduced with E6, we compared the TP53 expression levels in these cells with those in BMSCs and LIPO-863-B and LP6 cells (Supplementary Fig. 5). Similar *TP53* mRNA and TP53 protein expression levels were observed between 2H and 5H cells expressing LacZ or only CDK4 and BMSCs (Supplementary Fig. 5). It is noteworthy that both 2H and 5H cells expressing MDM2 and/or CDK4 showed decreased TP53 expression levels (Supplementary Fig. 5B). These data suggest that MDM2 overexpression can induce TP53 degradation in the presence of E6 in these cells.

**Fig. 3:**
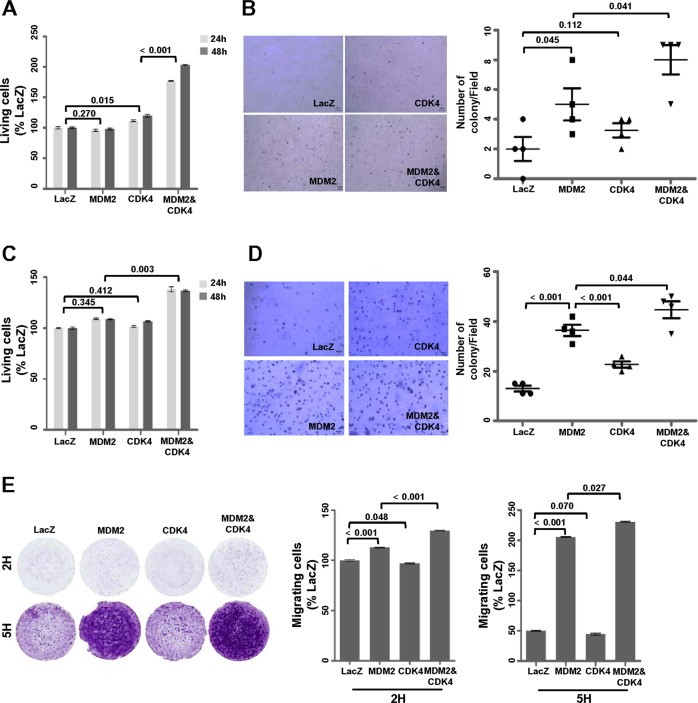
Co-overexpression of MDM2 and CDK4 synergistically promotes tumorigenic features. **a**, **c** Cell viability was evaluated via a WST-1 assay. **b**, **d** Anchorage-independent cell growth was analyzed via a soft agar assay. Images of cells expressing the indicated protein levels in agarose are shown (left panels), and the number of colonies/fields was counted by microscopy (right panels). 2H cells (**a**, **b**) and 5H cells (**c**, **d**). **e** Migration assay was performed in a transwell chamber. Images of crystal violet-stained cells expressing the indicated protein on membranes are shown in the left panels. Intensity was obtained by ultraviolet spectrometry, and the percentage values were determined based on LacZ cell intensity (right panels). *P-*values are presented for the indicated comparisons

Next, we examined whether the co-overexpression of MDM2 and CDK4 accelerates the tumorigenic potential in transformed cells. In both 2H and 5H cells, co-overexpression of MDM2 and CDK4 significantly increased cell proliferation (Fig. 3a, c), anchorage-independent cell growth (Fig. 3b, d), and cell migration (Fig. 3e) when compared with the sole expression of MDM2 or CDK4. Interestingly, 5H cells only expressing MDM2 showed significantly increased anchorage-independent cell growth (Fig. 3d) and activated cell mobility (Fig. 3e) but not cell proliferation (Fig. 3c), relative to the cells expressing CDK4 alone. These results indicate that the co-overexpression of MDM2 and CKD4 plays a key role in tumorigenesis in transformed BMSCs.

### Co-overexpression of MDM2 and CDK4 blocks the potential of adipogenic differentiation

**Fig. 4:**
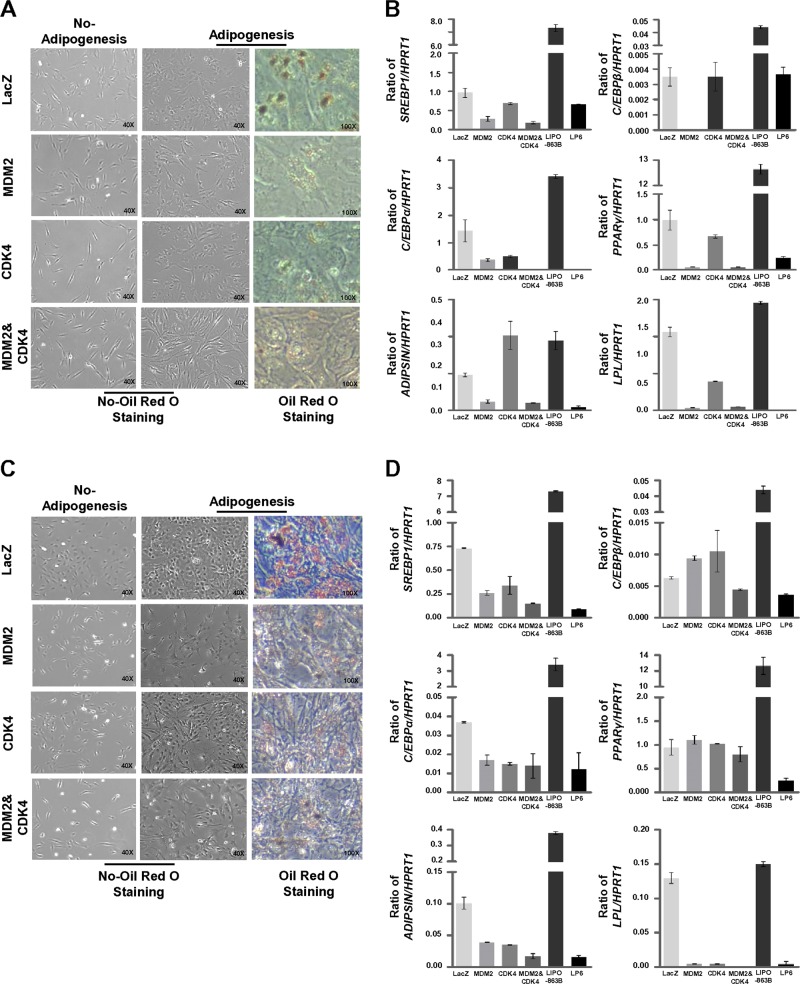
Co-overexpression of MDM2 and CDK4 blocks differentiation during the complete adipogenic process. **a**, **c** The ability to differentiate into adipocytes was evaluated by Oil Red O staining. **b**, **d** mRNA expression levels of *SREBP1*, *C/EBPβ*, *C/EBPα*, *PPARγ*, *ADIPSIN*, and *LPL* were quantified by quantitative RT-PCR after the induction of adipogenesis for 21 days. The ratio of the expression levels of the genes to those of *HPRT1* was used to determine the relative levels of all genes. 2H cells (**a**, **b**) and 5H cells (**c**, **d**)

To examine whether overexpression of MDM2 and CDK4 alters the adipogenesis potential, we first performed Oil Red O staining after culturing the cells in the adipogenic induction medium. 2H-MDM2 and CDK4 cells displayed small amounts of lipid droplets relative to 2H-LacZ cells and were longer and thinner than the cells expressing only MDM2 or CDK4 (Fig. 4a). 5H-MDM2 and 5H-MDM2 and CDK4 cells showed a reduced positivity of Oil Red O staining and contained stellar cell bodies, relative to both 5H-LacZ and 5H-CDK4 cells (Fig. 4c). Next, we analyzed the expression levels of genes serially induced during adipogenesis by real-time PCR: *C/EBPβ* (in the early step), *C/EBPα*, and *PPARγ* (from the middle to late steps), *C/SREBP1* (full step), and *ADIPSIN* and *LPL* (late step) [29]. As shown in Fig. 4b, cells expressing only MDM2 or co-overexpressing MDM2 and CDK4 showed relatively downregulated expression levels of these genes in comparison with those in both 2H-LacZ and 2H-CDK4 cells; the expression levels of all genes except *C/EBPβ* in these cells were similar to those in the LP6 cells. Compared with 5H-LacZ cells, MDM2 and/or CDK4 expression led to the decreased expression of all genes, except *C/EBPβ* and *PPARγ*. Notably, co-overexpression of MDM2 and CDK4 decreased the expression of all adipogenesis-related genes, showing levels similar to those in LP6 cells (Fig. 4d). Collectively, these data suggest that co-expression of MDM2 and CDK4 blocks the differentiation of adipogenesis from the early to late stages.

### Co-overexpression of MDM2 and CDK4 in transformed human BMSCs results in the development of proliferative sarcoma with a dedifferentiated liposarcoma-like morphology in vivo

**Fig. 5:**
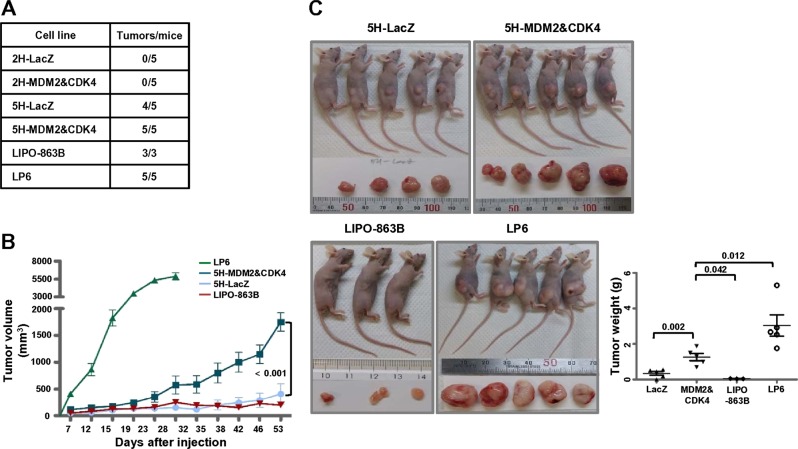
Co-expression of MDM2 and CDK4 in 5H cells causes increased tumor formation in vivo. **a** Incidence of tumor formation is illustrated. **b** Tumor volumes were measured using a digital caliper on the indicated day. **c** All xenografts were resected, and tumor weights were measured at the time of tumor harvest. *P-*values are presented for the indicated comparisons

To verify the in vivo tumorigenic potential of the transformed BMSCs, nude mice were subcutaneously inoculated with 2H and 5H cells co-overexpressing MDM2 and CDK4. The 2H cells did not develop into tumors, regardless of the co-expression of MDM2 and CDK4 (Fig. 5a). Consistent with the results of a study by Rodriguez et al., 5H-LacZ cells formed tumors with high penetrance (4/5, Fig. 5a) [19]. However, the 5H-MDM2 and CDK4 cells developed tumors larger than those observed in the LacZ control cells (Fig. 5b, c). In addition, LP6 cells showed a much more aggressive tumor formation in vivo than the 5H-MDM2 and CDK4 cells, despite their low growth potency in vitro (Fig. 5b, c; Supplementary Fig. 6).

**Fig. 6:**
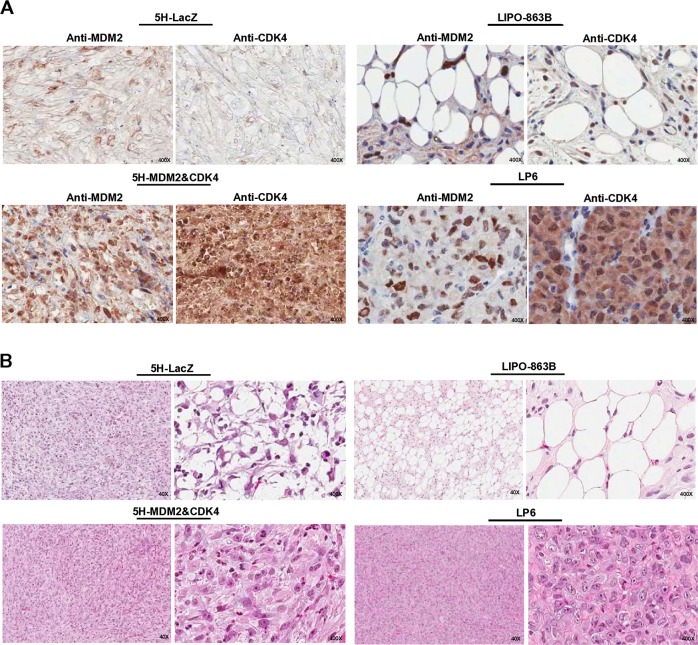
Co-expression of MDM2 and CDK4 in 5H cells induces proliferative DDLPS phenotypes in vivo. **a** The expression of MDM2 or CDK4 was examined by immunohistochemical staining in tumors developed from 5H-LacZ, 5H-MDM2 and CDK4, LIPO-863B, and LP6 cells. The original magnification is indicated. **b** Histological characteristics of the derived tumors were monitored by hematoxylin–eosin staining

Next, we performed the histological analysis of the 5H cell-derived tumors, including those from the LIPO-863 and LP6 xenograft models. The 5H-MDM2 and CDK4 cell-derived tumors were immunostained and found to be strongly positive for MDM2 and CDK4, while the tumors derived from 5H-LacZ cells were not (Fig. 6a). Although relatively high background was observed, their intensity levels were similar to those of LP6 cell-derived tumors (Fig. 6a). The 5H-MDM2 and CDK4 cell-derived tumors exhibited more proliferative features with high cellularity and higher expression of Ki-67 than did the 5H-LacZ-derived tumors (Fig. 6b; Supplementary Fig. 7). Moreover, these tumors morphologically resembled LP6 cell-derived tumors, displaying large nuclear cells of variable sizes dispersed within a fibrous matrix, but not LIPO-863B cell-derived tumors, which were composed of mature adipocytic cells of diverse sizes and associated with a variable number of atypical stromal cells (Fig. 6b; Supplementary Fig. 8). In addition, tumors from 5H-MDM2 and CDK4 cells showed a small proportion of lipoblast cells, but they were not immunostained with KU80, which has been reported as a marker of human cells; therefore, these lipoblast cells may not be derived from 5H-MDM2 and CDK4 cells (Supplementary Fig. 9) [30]. These findings indicate that co-overexpression of MDM2 and CDK4 in 5H cells with five additional oncogenic mutations can result in the development of proliferative sarcoma with a DDLPS-like morphology in vivo.

## Discussion

WDLPS/DDLPS is characterized by the amplification and overexpression of MDM2 and CDK4. However, several other oncogenes, such as HMGA2, c-JUN, ZIC1, etc., have been reported to contribute to the tumorigenesis and progression of WDLPS/DDLP [3, 4, 24, 31–34]. Although amplification and overexpression of MDM2 and CDK4 are hallmark events of WDLPS/DDLPS, whether MDM2 and CDK4 drive WDLPS/DDLPS tumorigenesis remains unclear. Therefore, it is necessary to evaluate whether the amplification and overexpression of MDM2 and CDK4 are critical events for WDLPS/DDLPS development. We addressed this question by establishing a corresponding xenograft.

Human BMSCs have not been shown to undergo spontaneous transformation in vitro, with the exception of a few cases in which BMSC-injected patients later developed osteosarcoma [35–38]. In addition, sarcomagenesis models expressing the fusion proteins EWS-FLI1 or SYT-SSX1 in human BMSCs failed to generate tumor phenotypes [17, 18]. However, the genetic introduction of tumor-suppressor genes such as TP53 and RB, and other oncogenes, such as the SV40 T antigen and HRAS, promoted BMSC transformation [39]. Rodriguez et al. first succeeded in inducing myxoid liposarcoma using BMSCs expressing the FUS-CHOP fusion protein and transformation with five oncogenic hits: TP53 deficiency, RB deficiency, hTERT overexpression, C-MYC stabilization, and HRAS^v12^ overexpression [19]. Thus, cooperating oncogenic hits are needed to transform BMSCs. Based on these reports, we tried to induce WDLPS/DDLPS in vivo by co-overexpressing MDM2 and CDK4 in transformed BMSCs.

According to a study by Taylor et al., *HDAC1*, *MAPKAP1*, *PTPN9*, and *DAZAP2* were mutated in DDLPS tissue samples by next-generation sequencing analysis [40]. Kanojia et al. found that *TERT*, *MCL1*, *ROR1*, *ERBB4*, *VEGFA*, *CPM2*, *ERBB3*, *SOCS2*, *CCNE1*, and *RUNX1* were amplified; *E2F6*, *CDKN2A*, and *NF1* were deleted; and *TP53*, *PLEC*, *FAT3*, *MXRAS*, *CHEK2*, and *NF1* were significantly altered in WDLPS/DDLPS tissue samples from copy number profiling (SNP array) analysis and targeted exome sequencing [41]. Notably, Ballinger et al. identified pathogenic germline mutations in 638 (55%) samples among 1162 sarcoma patients, using a targeted exon sequencing panel comprising 72 genes (based on associations with increased cancer risk), including *TP53* and *RB* [42]. They also showed that multiple mutations are significantly correlated with poorer tumor-free survival. Here, 2H cells with two oncogenic hits, TERT and HPV-16 E6 (TP53 degradation), did not develop into tumors, while 5H cells harboring three additional oncogenic hits, HPV-16 E7 (RB family inactivation), SV40-ST (c-MYC stabilization), and HRAS^v12^ (mitogenic signal activation), generated tumors in vivo. Our findings suggest that TERT and HPV-16 E6 may not cooperate with MDM2 and CDK4 for BMSCs to develop into WDLPS/DDLPS, but HPV-16 E7, SV40-ST, or HRAS^v12^ may be required to contribute to the transformation of BMSCs.

Our study has several limitations including that actual patient samples were not used to evaluate the expression levels of MDM2 and/or CDK4 in transduced 2H and 5H cells, and none of the other genes such as *HMGA2* and *CHOP* in the 12q13-15 amplicon were examined. Although the histologically analyzed 5H-MDM2 and CDK4-derived tumors could not be clearly classified as DDLPS, these tumors morphologically resembled LP6 cell-derived tumors, displaying large nuclear cells of variable sizes dispersed within a fibrous matrix. Thus, our model showed that co-expression of MDM2 and CDK4 in transformed human BMSCs increases the tendency of high-grade sarcoma with a DDLPS-like morphology. This will contribute to understanding the intermediate step for DDLPS development. MDM2 and CDK4 overexpression through gene amplification is an early event in liposarcoma tumorigenesis. However, it was reported that increased *MDM2* amplification was closely associated with histological grade in liposarcomas [43]. We previously reported that the high *CDK4* amplification group exhibited significantly poorer prognosis relative to the low *CDK4* amplification group in human WDLPS and DDLPS [22]. Moreover, DDLPS components generally showed higher expression levels of MDM2 and CDK4 than did paired WDLPS components from the same patients, although no significant correlation was revealed in amplification status of *CDK4* or *MDM2* [44]. Thus, co-overexpression of MDM2 and CDK4 might play a key role in tumorigenicity during the transformation of BMSCs after cooperation with multiple genetic alterations. DDLPS had been thought to develop from WDLPS after a long duration, and these viewpoints have been re-established by the report of the presence of exclusively low-grade dedifferentiated components with a specific genomic profile relative to WDLPS and the fact that most cases of DDLPS occur de novo (90%) [45–47]. Therefore, DDLPS has now been identified in the absence of WDLPS. Moreover, our in vivo experiments showed that DDLPS tumor potency may be induced without the WDLPS component. To comprehensively understand the mechanism of WDLPS/DDLPS development, the characterization of both germline and somatic genetic alterations is needed using a massive next-generation sequencing approach in different large cohorts of WDLPS or DDLPS.

Although cell proliferation and differentiation are regarded as mutually exclusive events, cross-talk has been reported between both processes during adipogenesis [48]. Previous reports have suggested that MDM2 promotes adipocyte differentiation through *CREB*-dependent transactivation or *CREB*-regulated transcriptional coactivator-mediated activation of STAT6 using mouse embryonic fibroblasts and mouse preadipocyte cells, and that CDK4 participates in adipogenesis through *PPARγ* activation [49–51]. However, Peng et al. showed that WDLPS/DDLPS cell lines exhibited low or negative levels of Oil Red O positivity and PPARγ relative to pre-adipocytes and adipocytes [23]. We also found that 2H-MDM2 and CDK4 and 5H-MDM2 and CDK4 cells showed a reduced positivity of Oil Red O staining, and co-overexpression of MDM2 and CDK4 decreased the expression of all adipogenesis-related genes. Therefore, MDM2 and/or CDK4 may function as initiating oncogenes to block adipogenic differentiation during WDLPS/DDLPS development.

In summary, co-overexpression of MDM2 and CDK4 causes high-grade sarcoma with a DDLLPS-like morphology in transformed human BMSCs by accelerating cell growth and migration, and the blockage of adipogenic potential, after cooperation with multiple genetic factors.

## Supplementary information

Supplementary Figure 1–9

Supplemental Figure Legends

Supplemental Table1
