# Supplementary material for: Co-expression of MDM2 and CDK4 in transformed human mesenchymal stem cells causes high-grade sarcoma with a dedifferentiated liposarcoma-like morphology
Source: Lab Invest. 2019 Jun 3;99(9):1309–20. doi: 10.1038/s41374-019-0263-4 (PMC6760642; doi:10.1038/s41374-019-0263-4)
Supplement: Supplementary file 1 — Supplementary Figure 1–9 [file 41374_2019_263_MOESM1_ESM.pptx]

## Slide 1
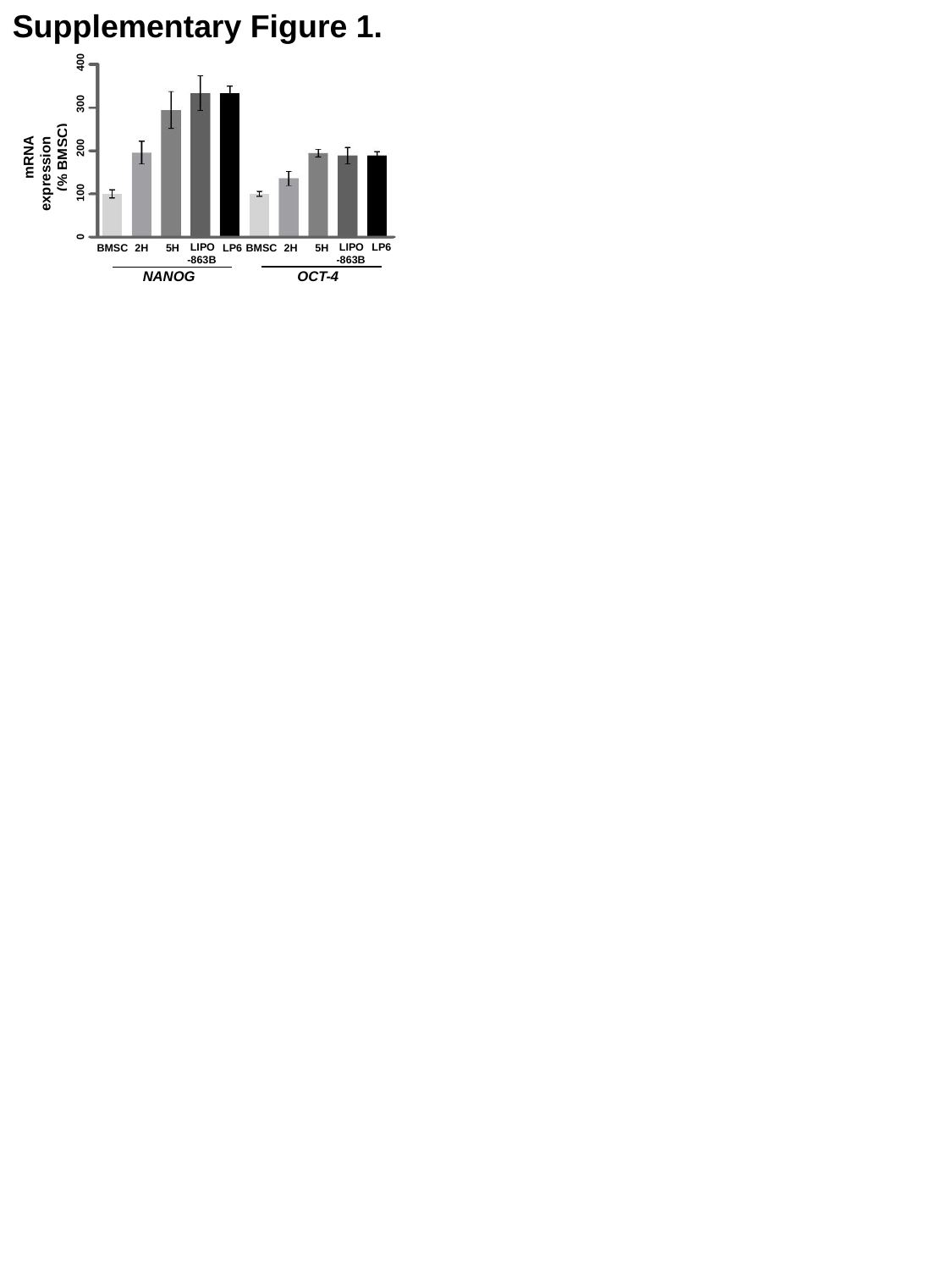

Supplementary Figure 1.
mRNA expression (% BMSC)
 0 100 200 300 400
 LP6
 LP6
 BMSC
 2H
 5H
 BMSC
 2H
 5H
 LIPO
 -863B
 LIPO
 -863B
OCT-4
NANOG

## Slide 2
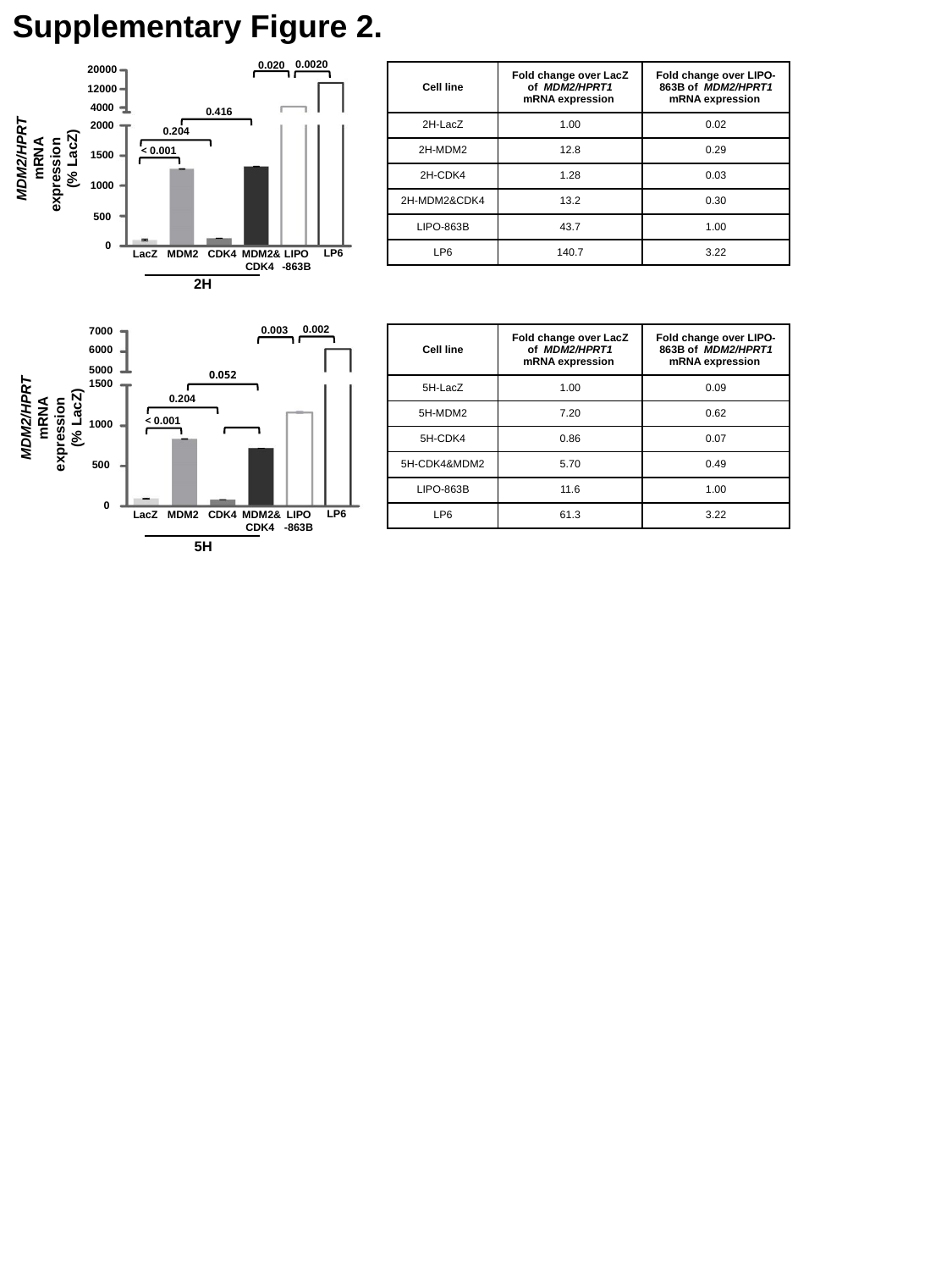

Supplementary Figure 2.
 0.0020
 0.020
 20000
| Cell line | Fold change over LacZ of MDM2/HPRT1 mRNA expression | Fold change over LIPO-863B of MDM2/HPRT1 mRNA expression |
| --- | --- | --- |
| 2H-LacZ | 1.00 | 0.02 |
| 2H-MDM2 | 12.8 | 0.29 |
| 2H-CDK4 | 1.28 | 0.03 |
| 2H-MDM2&CDK4 | 13.2 | 0.30 |
| LIPO-863B | 43.7 | 1.00 |
| LP6 | 140.7 | 3.22 |
 12000
 4000
 0.416
 2000
MDM2/HPRT
mRNA expression (% LacZ)
0.204
< 0.001
 1500
 1000
 500
 0
 LP6
 LacZ
MDM2
 CDK4
 MDM2&
CDK4
LIPO
-863B
2H
 0.002
 0.003
 7000
| Cell line | Fold change over LacZ of MDM2/HPRT1 mRNA expression | Fold change over LIPO-863B of MDM2/HPRT1 mRNA expression |
| --- | --- | --- |
| 5H-LacZ | 1.00 | 0.09 |
| 5H-MDM2 | 7.20 | 0.62 |
| 5H-CDK4 | 0.86 | 0.07 |
| 5H-CDK4&MDM2 | 5.70 | 0.49 |
| LIPO-863B | 11.6 | 1.00 |
| LP6 | 61.3 | 3.22 |
 6000
 5000
0.052
 1500
MDM2/HPRT
mRNA expression (% LacZ)
0.204
< 0.001
 1000
 500
 0
 LP6
 LacZ
MDM2
 CDK4
 MDM2&
CDK4
LIPO
-863B
5H

## Slide 3
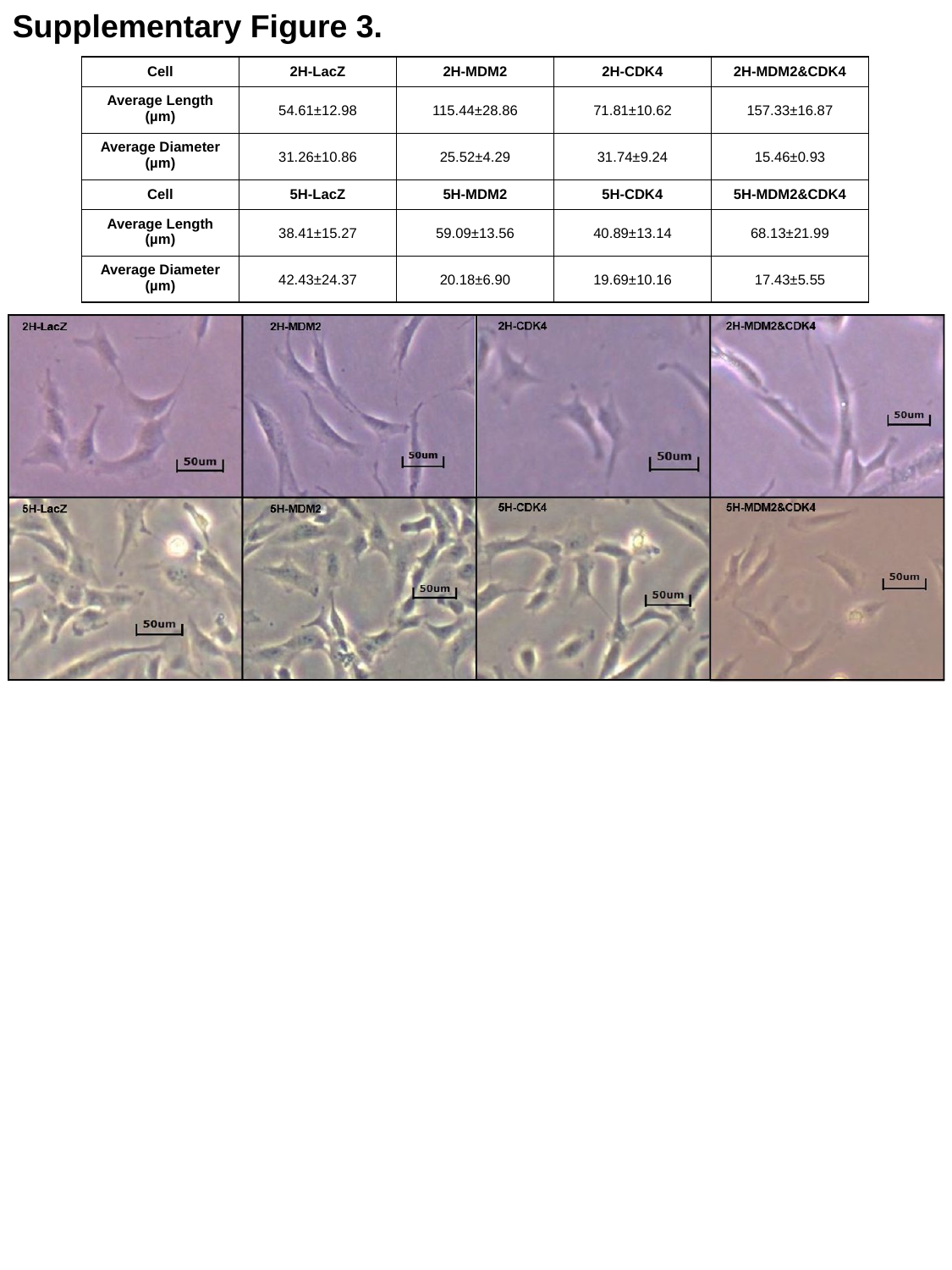

Supplementary Figure 3.
| Cell | 2H-LacZ | 2H-MDM2 | 2H-CDK4 | 2H-MDM2&CDK4 |
| --- | --- | --- | --- | --- |
| Average Length (µm) | 54.61±12.98 | 115.44±28.86 | 71.81±10.62 | 157.33±16.87 |
| Average Diameter (µm) | 31.26±10.86 | 25.52±4.29 | 31.74±9.24 | 15.46±0.93 |
| Cell | 5H-LacZ | 5H-MDM2 | 5H-CDK4 | 5H-MDM2&CDK4 |
| Average Length (µm) | 38.41±15.27 | 59.09±13.56 | 40.89±13.14 | 68.13±21.99 |
| Average Diameter (µm) | 42.43±24.37 | 20.18±6.90 | 19.69±10.16 | 17.43±5.55 |

## Slide 4
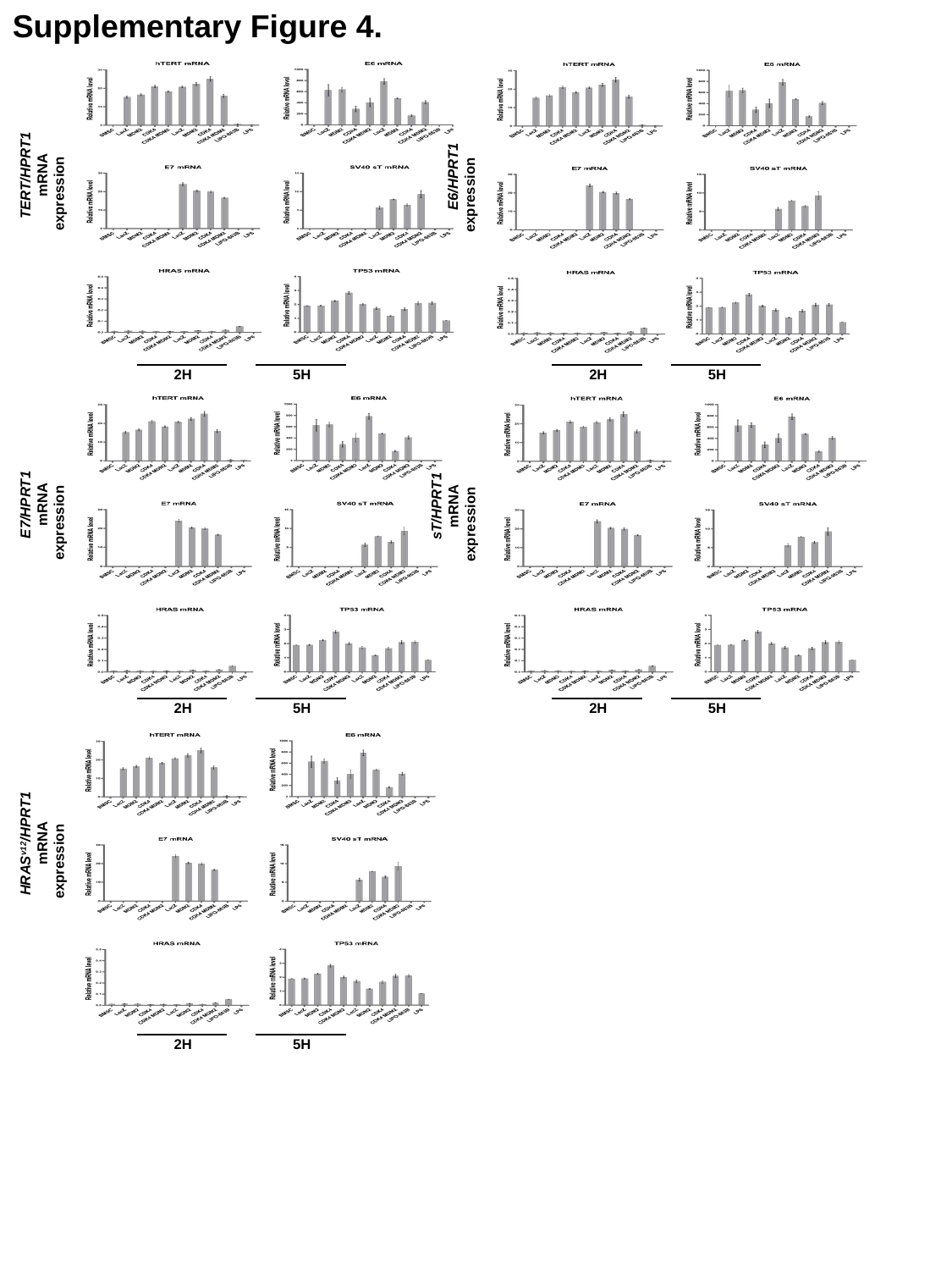

Supplementary Figure 4.
TERT/HPRT1
mRNA expression
E6/HPRT1
expression
2H
5H
2H
5H
E7/HPRT1
mRNA expression
sT/HPRT1
mRNA expression
2H
5H
2H
5H
HRASv12/HPRT1
mRNA expression
2H
5H

## Slide 5
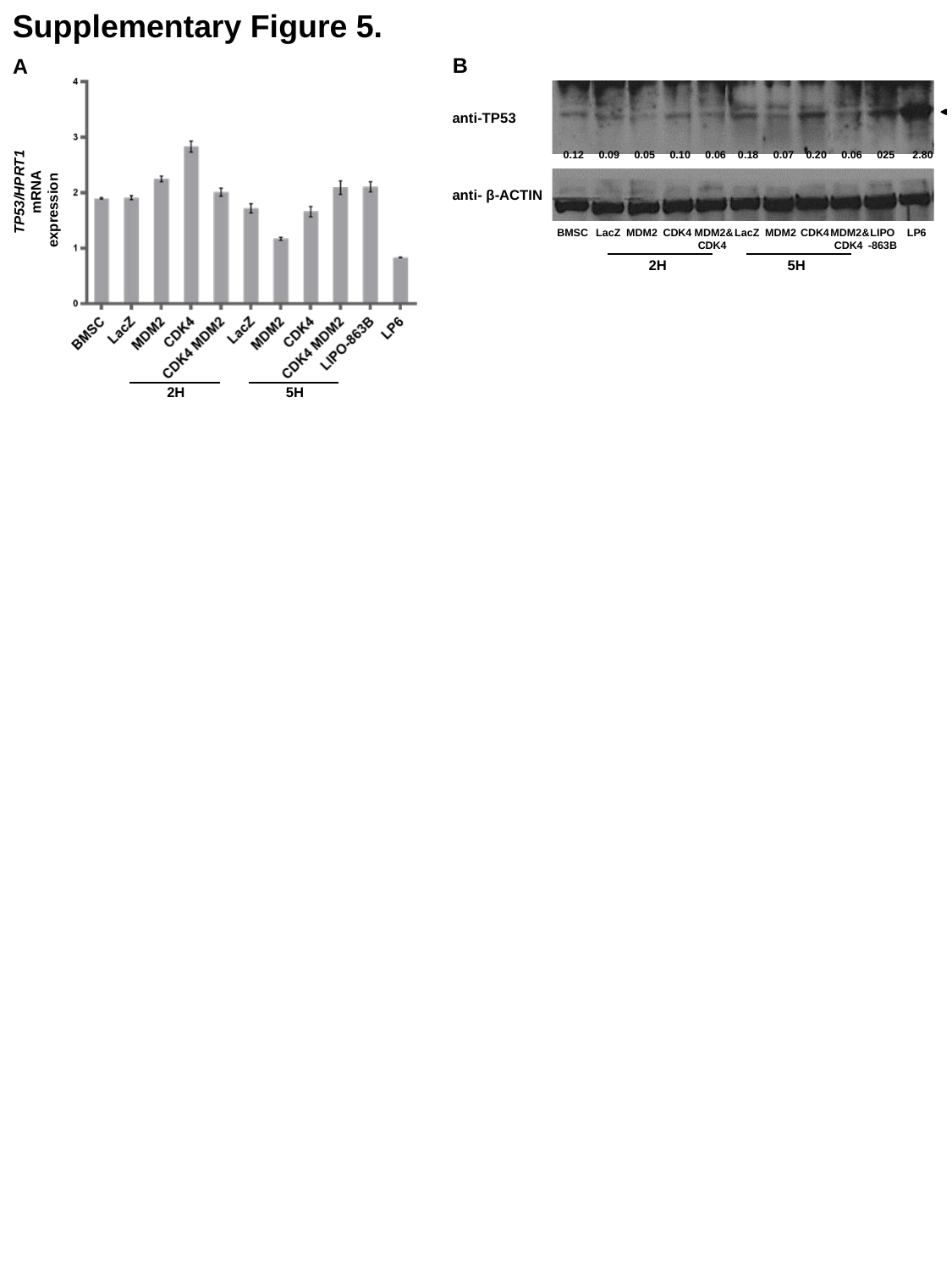

Supplementary Figure 5.
B
A
 anti-TP53
 0.12 0.09 0.05 0.10 0.06 0.18 0.07 0.20 0.06 025 2.80
TP53/HPRT1
mRNA expression
 anti- β-ACTIN
 BMSC
 LacZ
MDM2
 CDK4
 LacZ
MDM2
 CDK4
 LP6
 MDM2&
CDK4
 MDM2&
CDK4
LIPO
-863B
2H
5H
2H
5H

## Slide 6
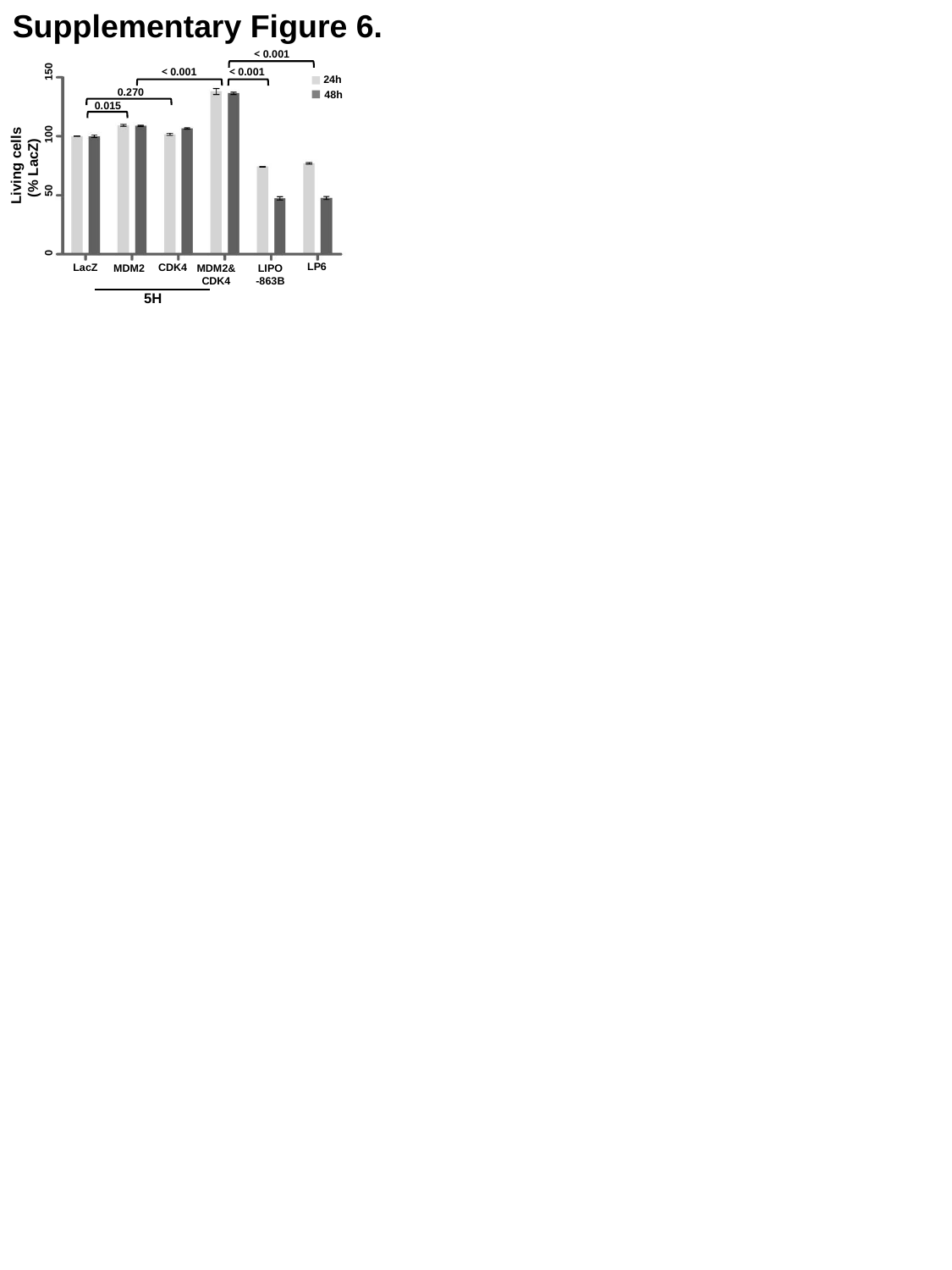

Supplementary Figure 6.
< 0.001
< 0.001
< 0.001
24h
0.270
48h
0.015
 0 50 100 150
Living cells
(% LacZ)
LP6
LacZ
CDK4
MDM2
LIPO
-863B
MDM2&CDK4
5H

## Slide 7
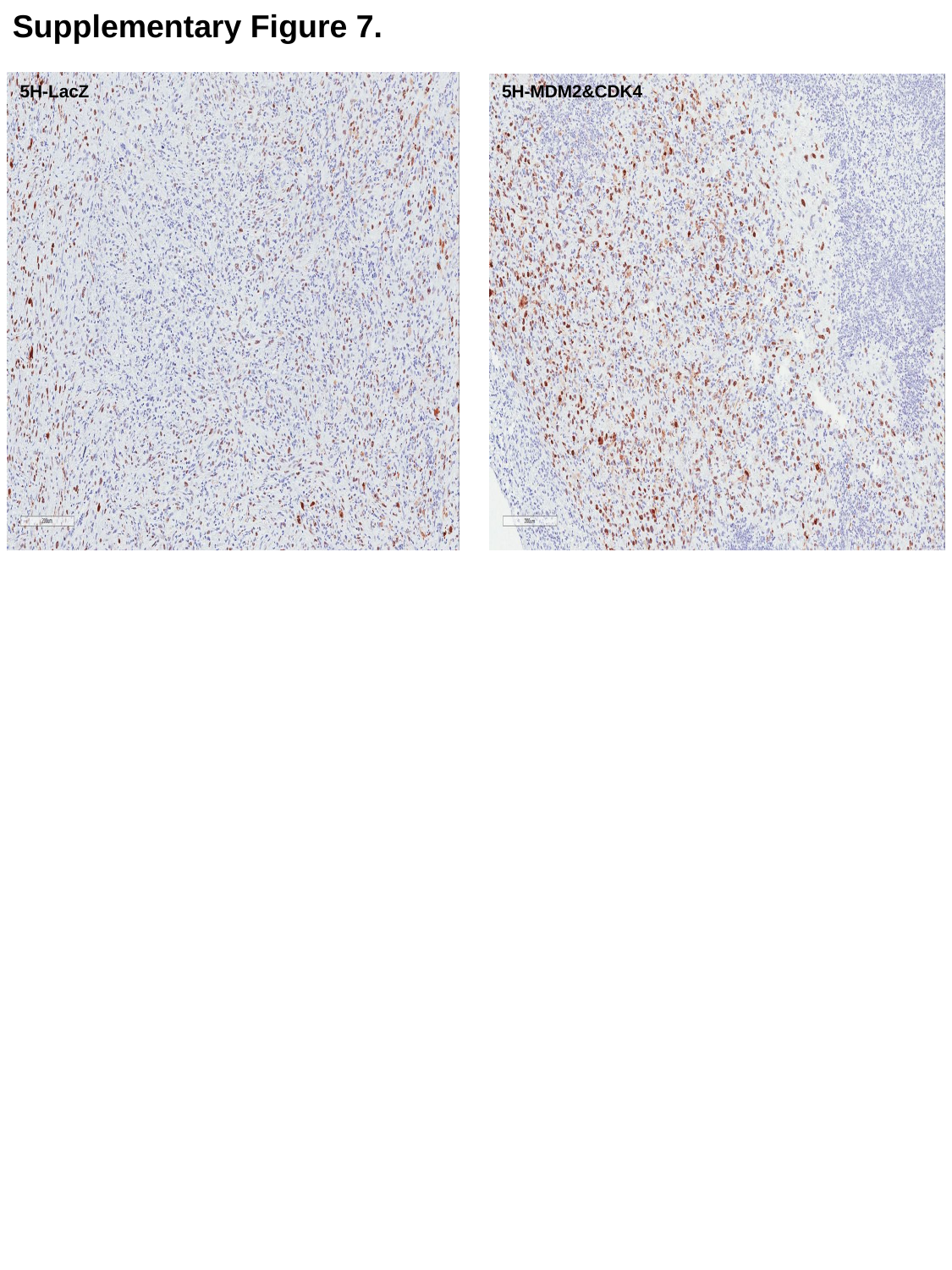

Supplementary Figure 7.
5H-MDM2&CDK4
5H-LacZ

## Slide 8
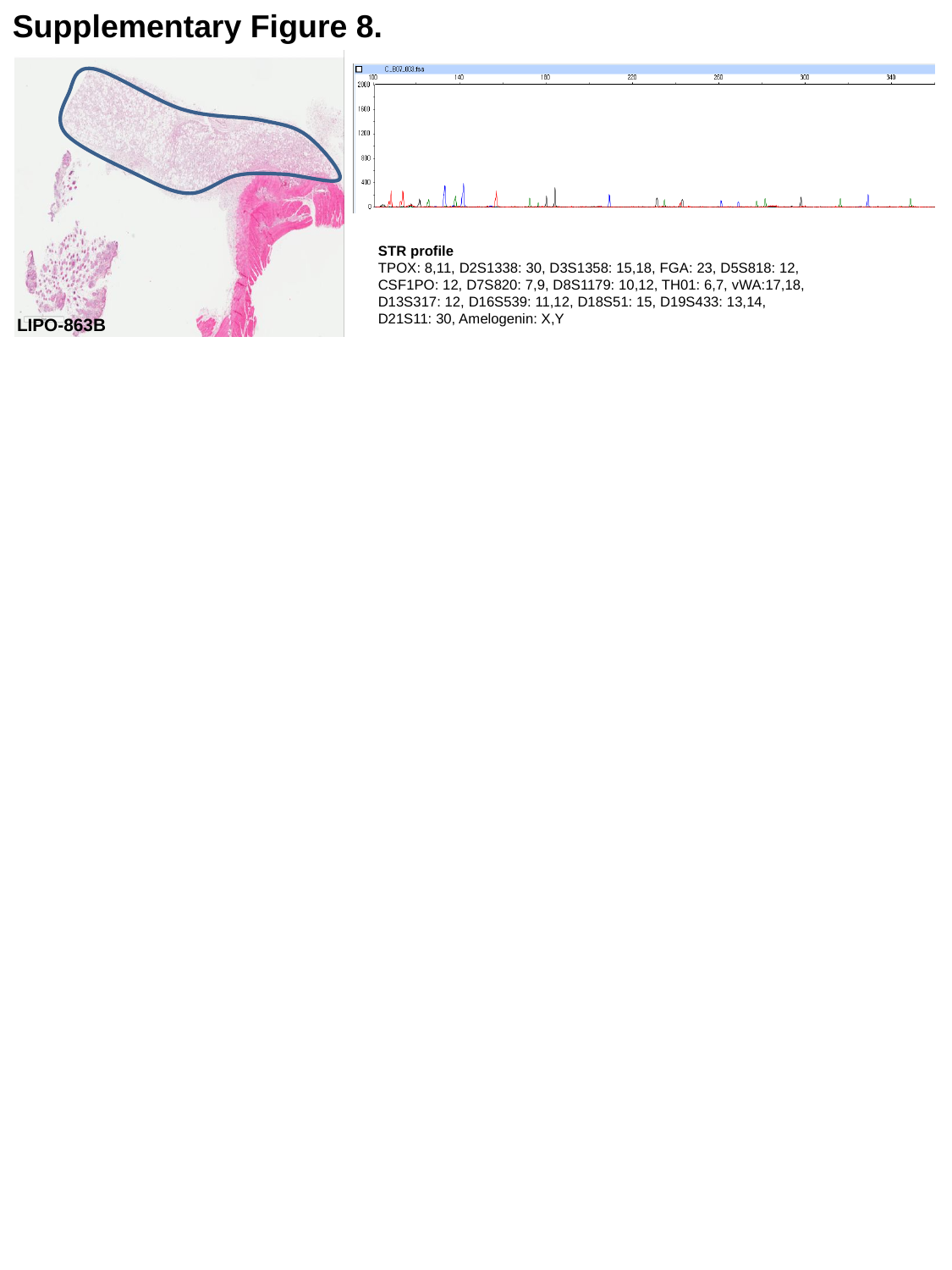

Supplementary Figure 8.
STR profile
TPOX: 8,11, D2S1338: 30, D3S1358: 15,18, FGA: 23, D5S818: 12, CSF1PO: 12, D7S820: 7,9, D8S1179: 10,12, TH01: 6,7, vWA:17,18,
D13S317: 12, D16S539: 11,12, D18S51: 15, D19S433: 13,14,
D21S11: 30, Amelogenin: X,Y
LIPO-863B

## Slide 9
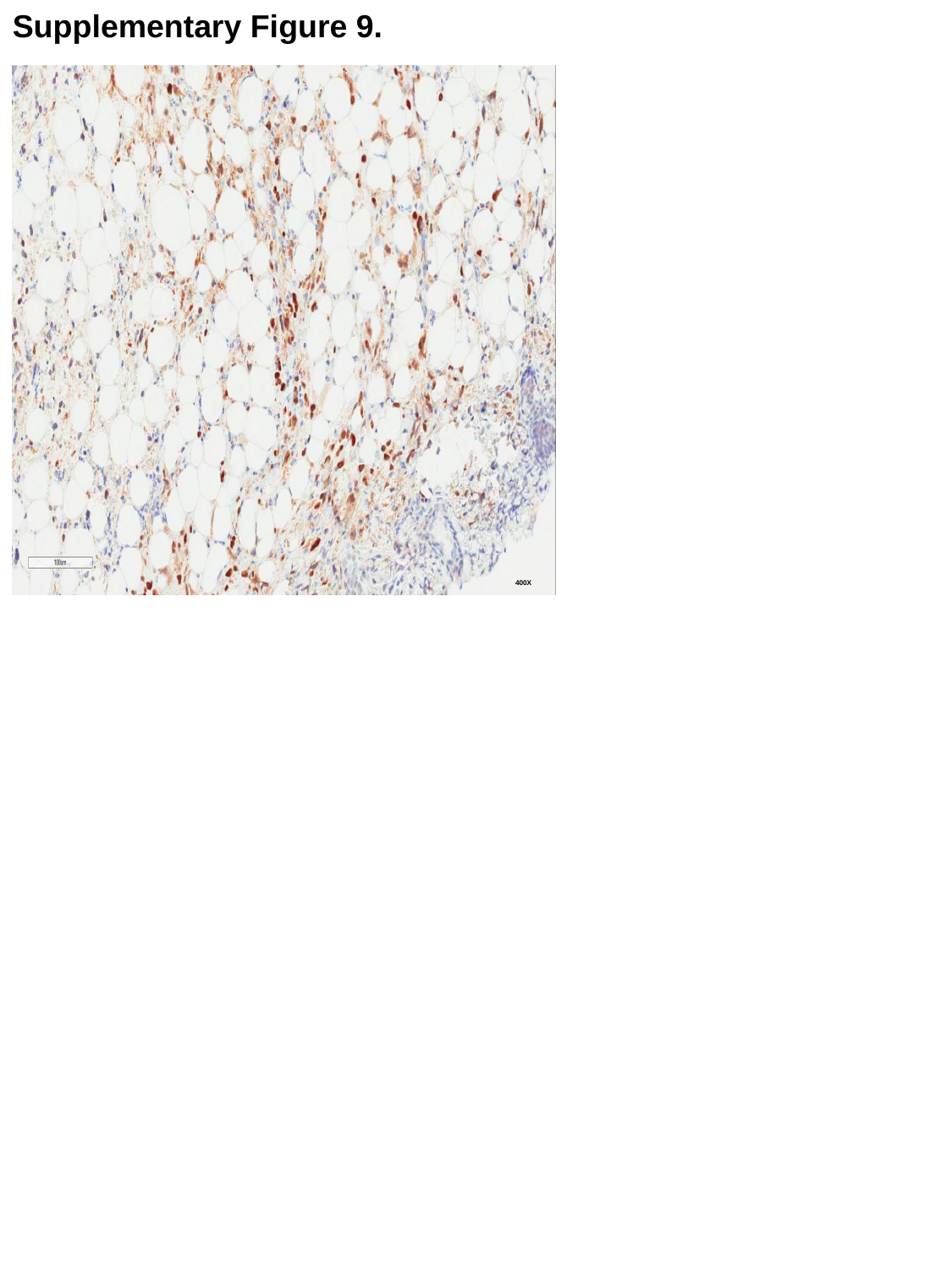

Supplementary Figure 9.
 400X
