# Supplementary material for: Co-expression of MDM2 and CDK4 in transformed human mesenchymal stem cells causes high-grade sarcoma with a dedifferentiated liposarcoma-like morphology
Source: Lab Invest. 2019 Jun 3;99(9):1309–20. doi: 10.1038/s41374-019-0263-4 (PMC6760642; doi:10.1038/s41374-019-0263-4)
Supplement: Supplementary file 2 — Supplemental Figure Legends [file 41374_2019_263_MOESM2_ESM.docx]

**Supplementary figure legends for Co-expression of MDM2 and CDK4 in transformed human mesenchymal stem cells causes high-grade sarcoma with a dedifferentiated liposarcoma-like morphology**

**Supplementary Figure 1.** mRNA expression levels of *NANOG* and *OCT-4* in DMEM were quantified by quantitative RT-PCR. The ratio of the expression levels of the genes to those of *HPRT1* was used to determine the relative levels of all genes. The percentage values were calculated based on their levels in BMSCs.

**Supplementary Figure 2.** mRNA expression of *MDM2* was measured by quantitative RT-PCR. Fold-changes were determined by comparing the levels expressed in LacZ and LIPO-863B cells.

**Supplementary Figure 3.** Average length and diameter were determined by Infinite Analyze software in 2H and 5H cells stably expressing MDM2 and/or CDK4.

**Supplementary Figure 4.** mRNA expression levels of *TERT*, *E6*, *E7*, *sT*, and *HRAS^v12^* were measured using quantitative RT-PCR. The ratio of the expression levels of the genes to those of *HPRT1* was used to determine the relative levels of all genes.

**Supplementary Figure 5.** (A) mRNA expression of *TP53* was measured using quantitative RT-PCR. The ratio of the expression levels of *TP53* to those of *HPRT1* was used to determine the relative levels of *TP53.* (B) The protein expression of TP53 was measured by immunoblotting. ◀, TP53. Fold-changes were determined by comparing the protein levels to the β-ACTIN levels using ImageJ.

**Supplementary Figure 6.** Cell viability was evaluated by the WST-1 assay. The *P* values are presented for the indicated comparisons.

**Supplementary Figure 7.** Expression of Ki-67 was examined by the immunohistochemical staining in 5H-LacZ and 5H-MDM2&CDK4 cell-derived tumors.

**Supplementary Figure 8.** STR profile was confirmed by the analysis of lipoblasts obtained through the dissection of LIPO-863B cell-derived tumors.

**Supplementary Figure 9.** Expression of KU80 was examined by the immunohistochemical staining of lipoblasts from 5H-MDM2&CDK4
