# Supplementary material for: Co-expression of MDM2 and CDK4 in transformed human mesenchymal stem cells causes high-grade sarcoma with a dedifferentiated liposarcoma-like morphology
Source: Lab Invest. 2019 Jun 3;99(9):1309–20. doi: 10.1038/s41374-019-0263-4 (PMC6760642; doi:10.1038/s41374-019-0263-4)
Supplement: Supplementary file 3 — Supplemental Table1 [file 41374_2019_263_MOESM3_ESM.docx]

**Supplementary Table 1.** Primer sets of qRT-PCR

| **Primer name** | **Primer sequence** |
| --- | --- |
| E6 antigen of HPV16_F | AGCGACCCAGAAAGTTACCA |
| E6 antigen of HPV16_R | GCATAAATCCCGAAAAGCAA |
| E7 antigen of HPV16_F | AGGAGGATGAAATAGATGGTCCAG |
| E7 antigen of HPV16_R | CTTTGTACGCACAACCGAAGC |
| HRAS_F | GCGCCTGTGAACGGTGG |
| HRAS_R | TGGGCACGTCATCCGAGTCC |
| hTERT_F | GGAGCAAGTTGCAAAGCATTG |
| hTERT_R | TCCCACGACGTAGTCCATGTT |
| SV40 sT_F | TGCAGCTAATGGACCTTCTAGGT |
| SV40 sT_R | GAATATTCCCCCAGGCACTC |
| TP53_F | CCGCAGTCAGATCCTAGCG |
| TP53_R | TGGGTCTTCAGTGAACCATTGT |
